# Anti-Inflammatory Mechanisms of Total Flavonoids from *Mosla scabra* against Influenza *A* Virus-Induced Pneumonia by Integrating Network Pharmacology and Experimental Verification

**DOI:** 10.1155/2022/2154485

**Published:** 2022-06-08

**Authors:** Wei Cai, Shui-Li Zhang

**Affiliations:** ^1^Department of Chinese Materia Medica, Zhejiang Pharmaceutical University, Ningbo 315100, China; ^2^Zhejiang Chinese Medical University, Hangzhou 310053, China

## Abstract

Influenza virus is one of the most common infectious pathogens that could cause high morbidity and mortality in humans. However, the occurrence of drug resistance and serious complications extremely complicated the clinic therapy. *Mosla scabra* is a natural medicinal plant used for treating various lung and gastrointestinal diseases, including viral infection, cough, chronic obstructive pulmonary disease, acute gastroenteritis, and diarrhoea. But the therapeutic effects of this herbal medicine had not been expounded clearly. In this study, a network pharmacology approach was employed to investigate the protective mechanism of total flavonoids from *M. scabra* (MSTF) against influenza *A* virus- (IAV-) induced acute lung damage and inflammation. The active compounds of MSTF were analyzed by LC-MS/MS and then evaluated according to their oral bioavailability and drug-likeness index. The potential targets of each active compound in MSTF were identified by using PharmMapper Server, whereas the potential genes involved in IAV infection were obtained from GeneGards. The results showed that luteoloside, apigenin, kaempherol, luteolin, mosloflavone I, and mosloflavone II were the main bioactive compounds found in MSTF. Primarily, 23 genes were identified as the targets of those five active compounds, which contributed to the inactivation of chemical carcinogenesis ROS, lipid and atherosclerosis, MAPK signaling pathway, pathways in cancer, PI3K-AKT signaling pathway, proteoglycans in cancer, and viral carcinogenesis. Finally, the animal experiments validated that MSTF improved IAV-induced acute lung inflammation via inhibiting MAPK, PI3K-AKT, and oxidant stress pathways. Therefore, our study demonstrated the potential inhibition of MSTF on viral pneumonia in mice and provided a strategy to characterize the molecular mechanism of traditional Chinese medicine by a combinative method using network pharmacology and experimental validation.

## 1. Introduction

Influenza is an acute viral respiratory infection caused by influenza viruses, which have high infectivity and incidence [1]. Influenza viruses are commonly classified into three types: *A*, *B,* and *C*. Due to the susceptibility of the influenza virus, it has caused many worldwide pandemics in decades, resulting in numerous deaths and huge economic losses [2, 3]. Also, this high variability of influenza viruses makes it more difficult for humans to cope with influenza. Since the development and vaccination of preventive vaccines cannot be targeted, the application of anti-influenza drugs has become one of the important strategies to prevent and treat influenza.

Chinese traditional medicine has unique advantages in the prevention of influenza viral infection and its secondary diseases [4–7]. Some herbal extracts have direct viral inhibitory effects and can play indirect antiviral effects by regulating the body's immune function, such as *Scutellariae Radix*, *Salviae miltiorrhizae Radix* et *Rhizoma*, *Lonicerae japonicae Flos*, lianhuaqingwen capsule, and other compound medicines [7–13]. Therefore, the extracted parts of traditional Chinese medicine with comprehensive antiviral effects have good development and application prospects.

*Mosla scabra*, belonging to *Lamiaceae* family, is a perennial herb that has been used for years in the treatment and prevention of lung and gastrointestinal diseases, including viral infection, cough, chronic obstructive pulmonary disease, acute gastroenteritis, and diarrhoea [14, 15]. Recently, many pharmacologists paid close attention to the various chemical constituents and biological activities. This herb is abundant in lignans, flavonoids, monoterpenoids, and diketopiperazines, which exerts antioxidant, anti-inflammatory, antiallergic, anticoagulant, and antibacterial effects [16–21]. Yu et al. reported that the ethyl acetate extract of *M. scabra* (MSAE) was the main active faction in the herbal medicine, which reduced the viral replication in vivo and in vitro as well as influenza virus-induced pneumonia and alveolar fluid dysfunction via repressing MAPK/AQP5 and NOX4/NF-*κ*B pathways [14, 22]. Further chemical indentation by LC-MS/MS showed that MSAE was an excellent source of flavonoids including mosloflavone I, mosloflavone II, luteolin, kaempferol, apigenin, and luteoloside; luteolin and kaempferol, especially, had played the major contribution for antiviral and anti-inflammatory effects of total flavonoids from *M. scabra* (MSTF) [22, 23]. It is well known that the pharmacodynamic effects of herbal medicine are determined by its mass active components. Although recent findings on antiviral mechanisms of MSTF had been progressively represented on influenza virus-induced microvascular and alveolar epithelial damage [22, 23], its therapeutic effects on influenza virus-induced lung microenvironment injury have not been elaborated completely. Therefore, in this study, to investigate the mechanism underlying overall therapeutic effects of MSTF against influenza virus-induced pneumonia in mice, the anti-inflammatory actions of five main bioactivity compounds (luteolin, kaempferol, apigenin, mosloflavone I, and mosloflavone II) in MSTF were evaluated by using a combinative method of network pharmacology analysis and experimental verification.

## 2. Materials and Methods

### 2.1. Preparation of MSTF

The extraction and purification of MSTF were performed as previously reported [15, 23]. Briefly, the dried plant materials (the aerial parts, 1.0 kg) were extracted with boiling water two times for 1 h. The mixed decoction was concentrated to 0.4 multiple of raw extracting solution and then extracted by ethyl acetate four times. The extract was vacuum evaporated to recover the solvent. The dried brown extracts were dissolved in the boiled water as the test drug (namely, MSTF) for further study. The contents of MSTF were quantitatively measured by colorimetric assay and HPLC [22]. HPLC experiments were performed on an Agilent 1200 HPLC system equipped with Hypersil BDS C18 (250 mm × 4.6 mm, 5 *μ*m) column, vacuum degasser, binary pump, and UV detector. The mobile phase was methanol (A) and 0.1% phosphoric acids (B) with a flow rate of 1 ml/min for gradient elution: 0–13 min, 30%⟶50% B; 13∼30 min, 50%⟶70% B; 30∼40 min, 70%⟶70%B. The column temperature was set at 30°C and detection wavelength at 270 nm. The samples were filtered through 0.45-*μ*m membrane before injection.

### 2.2. IAV-Infected Mouse Model and Drug Administration

The influenza A/PR/8/34 virus (IAV) was kept in Zhejiang Provincial Laboratory of Experimental Animal's & Nonclinical Laboratory Studies, and biosecurity procedures are strictly enforced in Animal Biosafety level 2 laboratory of Hangzhou Medical College. The infection was implemented under ether anesthesia by intranasal administration of 20 *μ*l IAV dilution (containing 7.9 × 10^6^ copies of IAV which was determined by qRT-PCR), which could cause the death of 70% mice.

Sixty ICR (Institute of Cancer Research) mice (20g–24 g body weights) were obtained from Zhejiang Experimental Animal Center. The experiments were approved by the ethics committee of Zhejiang Pharmaceutical Vocational University. The mice were randomly assigned to control group (Ctrl), model group, positive group (amantadine, 40 mg/kg), low-dose MSTF group, middle-dose MSTF group, and high-dose MSTF group. Sixty participants were balanced across the six groups. Except the mice in the control group, other mice were intranasally challenged with 100-fold dilution of the lung homogenates derived from the IAV-infected mice, which made 90% infected mice death. Two hours after infection, those infected mice were orally administrated with MSTF (50, 100, and 200 mg/kg, resp.) once a day for five days. The ribavirin was used as the positive control. Five days after viral infection, all the mice were killed after isoflurane anesthesia.

### 2.3. Serum Cytokine Analysis

Blood samples were collected and centrifuged at 3000 × *g* for 20 min to obtain the serum. The levels of proinflammatory cytokines IL-6 and IL-8 in serum were determined by using the ELISA kits (Biovol Technologies, Shanghai, China) according to the protocols.

### 2.4. Histopathology

The right lung was routinely fixed, embedded, sectioned, and stained with HE, and the histopathological changes of the lung were observed under light microscopy (Leica, Japan). The histological characteristics were scored by using the following grading scale: 0 (no), 1 (slight), 2 (moderate), and 3 (severe) [22]. Each part was individually assessed into neutrophil infiltration, alveolar congestion, and interstitial edema. The sum of those four indexes was recorded to display the severity of IAV-induced lung inflammation.

### 2.5. Real Time Fluorescent Quantitative PCR (RF-PCR) Analysis

The viral RNA in the lung tissues of IAV-infected mice was extracted according to the reagent instructions (Takara, Japan) and was used as the template for reverse transcription. The specific primer pairs were designed as follows: F 5′-AAATCT AGTGGT ACCGAG ATATGCA-3′, R: 5′-GGGAGG CTGGTG TTTATA GCAC-3′; the amplified product is about 173 bp as expected. The reaction conditions were 70°C for 10 min and 37°C for 90 min.

### 2.6. Western Blot Analysis

The prepared protein supernatant was quantified by the BCA method at 50 *μ*g per sample. The gel was electrophoresed at 60 V for concentration and 80 V for separation. The primary antibody (Santa Cruz, USA) was incubated in TBST with 5% skimmed milk powder for 1 h at a concentration of 1 : 1000 and incubated overnight at 4°C with gentle shaking. After incubation with primary antibody, the membrane was washed three times with TBST for 10 min, and secondary antibody (Santa Cruz, USA) was added at a concentration of 1 : 4000. The film was photographed, and the western blot images were analyzed by using GE imaging system. The ratio of the grey value of the target band to the grey value of *β*-actin was used to indicate the relative protein levels of the target proteins.

### 2.7. Network Pharmacology Analysis

The parameters, such as oral bioavailability (OB ≥ 20%) and drug-likeness (DL ≥ 0.18), of the main active compounds in MSTF were obtained from pharmacology database of the Chinese traditional medicine system TCMSP (https://old.tcmsp-e.com/tcmsp.php) [24]. The potential targets of the flavonoids were forecasted by PharmMapper Server (https://www.lilab-ecust.cn/pharmmapper/) [25]. “Influenza” was set as the keyword in GeneCards (https://www.genecards.org) database to gain the potential therapeutic targets of IAV-induced lung inflammation. The correlation among main compounds, targets, and pathways was analyzed by using LC omicstudio webservice.

### 2.8. Statistical Analysis

All values were expressed as mean values ± standard deviation (SD). All statistical analyses were performed using one-way ANOVA followed by the Turkey's post hoc test [26, 27]. *P* < 0.05 was considered statistically significant.

## 3. Results

### 3.1. Quality Evaluation of MSTF

**Figure 1:**
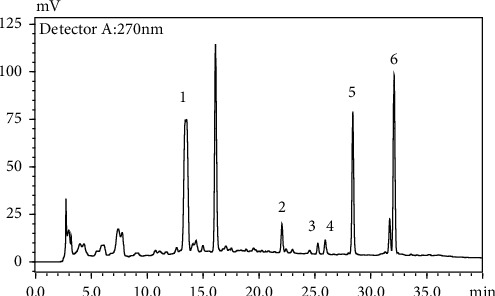
The HPLC chromatographic profiles of total flavonoids from *Mosla scabra*. The one-to-six peaks were mapped to the flavonoids as shown in Table 1.

Six marker compounds (luteoloside, luteolin, kaempferol, apigenin, mosloflavone I, and mosloflavone II) in MSTF shown in Table 1 were well identified by comparing their retention times and UV spectra with the standards by using LC-MS/MS analysis (Figure 1), whose contents in MSTF were 151.9, 74.1, 14.0, 26.8, 4.71, and 1.18 mg/g, respectively. This result was consistent with previous reports [22, 23].

### 3.2. Histological Analysis

**Figure 2:**
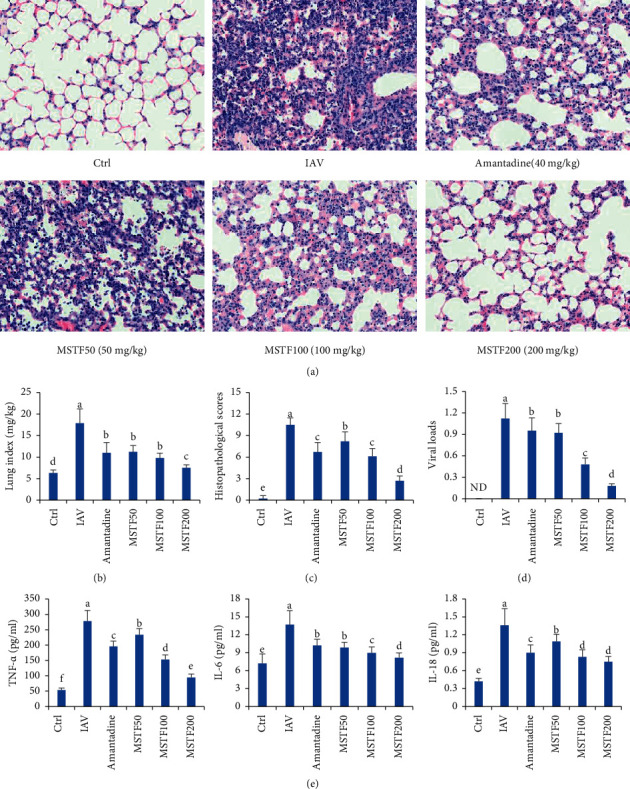
Effects of MSTF on IAV-induced lung inflammation. (a) Histopathological changes of lung tissues of each group, (b) inhibition of MSTF on lung indexes, (c) histopathological scores, (d) viral loads, and (e) serum levels of proinflammatory cytokines (TNF-*α*, IL-6, and IL-18) in IVA-infected mice. All data were shown as mean ± SEM (*n* = 10). Different letters meant significant differences (*P* < 0.05) by Tukey's test.

As shown in Figure 2, in the control group, alveolar structure was clear, and there were not any inflammatory cell infiltrations in the alveolar interstitial. After viral infection, lots of inflammatory cells were infiltrated in the alveolar interstitial, leading to the disappearance of alveolar structure as well as the increases of lung indexes and viral loads, which were much higher than those of the normal mice (*P* < 0.05). However, treatment with MSTF (50–200 mg/ml) could dose-dependently reduce the infiltration of inflammatory cells and blood cells, decrease the lung indexes, and lessen the viral copies in mice. More importantly, treatment with MSTF at the dose of 200 mg/kg presented better therapeutic effects than 40 mg/kg of amantadine. It indicated that MSTF improved IAV-induced lung injury and viral replication in mice.

### 3.3. Effects of MSTF on Inflammatory Cytokine Production

The results in Figure 2 revealed that the levels of IL-18, IL-6, and TNF-*α* in the serum of infected mice were 3∼4-fold higher than those of the control mice (*P* < 0.05). Administration with both MSTF and amantadine significantly reduced the serum levels of those proinflammatory cytokines (*P* < 0.05) and especially treatment with 100–200 mg/kg of MSTF exerted better inhibition on IAV-induced increase of serum IL-18, IL-6, and TNF-*α* levels than those of amantadine. It indicated that MSTF had the potential anti-inflammatory effects on IAV-induced lung inflammation in mice.

### 3.4. Protective Mechanism of MSTF against IAV-Induced Lung Pneumonia

**Figure 3:**
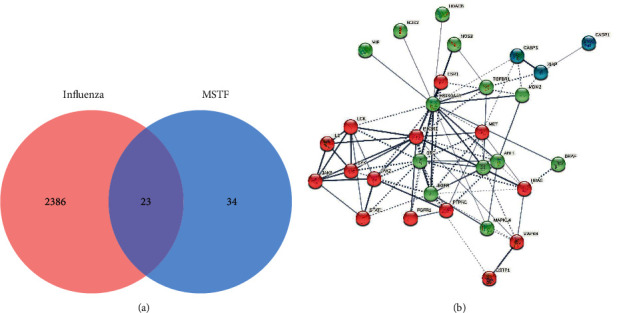
The target profiles of MSTF on IAV-induced lung inflammation. (a) Venn diagrams showed the overlap between influenza-related genes derived from GeneCards and MSTF targets predicted by PharmMapper Server. (b) Interactions among 23 overlapped targets of MSTF. The edges indicate both functional and physical protein associations; line thickness indicates the strength of data support.

The pharmacodynamic parameters of six main flavonoids identified by LC-MS/MS were calculated by using TCMSP service. Since luteoloside with low bioavailability could be broken down into luteolin and glucoses in the small intestine, luteolin, whose bioavailability was larger than 30%, was used to replace drug-target identification of luteoloside. Finally, 57 targets of those flavonoids were found, in which 23 targets were also in the collection of 2409 influenza-related genes obtained from GeneCards (Figure 3). There were close interactions among these 23 targets, which formed a complex gene network to regulate host antiviral response precisely. To explicit the functional distribution of 23 enriched targets, STRING was used to establish an interaction network among each target protein. All those targets could be divided into three main clusters (namely, inflammation, proliferation, and apoptosis).

**Figure 4:**
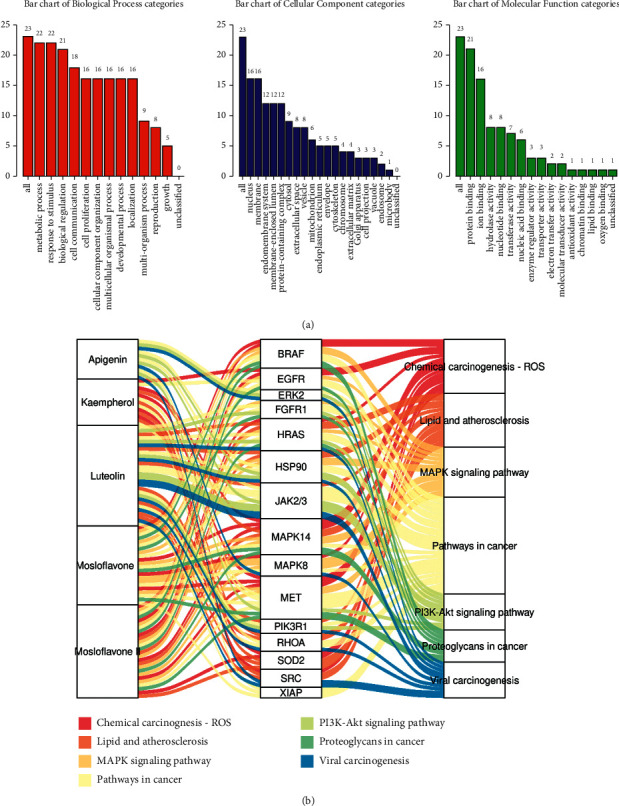
Identification and enrichment analysis of 23 targets of main flavonoids in MSTF. (a) The GO enrichment analysis of MSTF targets in treating IAV-induced lung inflammation. The *x*-axis was the enrichment degree, and the *y*-axis was the functional classification (i.e., biological process, cell components, and molecular function). (b) Sankey diagram showed relationships among five flavonoids, targets, and pathways. The curve thickness indicates the contribution to the regulation on the corresponding pathways.

The results of GO analysis showed that those targets primarily involved in the metabolic process, response to stimulus, and biological regulation via protein binding and ion binding patterns. The further KEGG results presented that those five flavonoids mainly targeted the main 15 proteins, such as MAPK14, MET, HRAS, HSP90, and JAK2/3, which involved mainly in regulating IAV-related signaling, including pathways in cancer, MAPK signaling pathway, lipid and atherosclerosis, chemical carcinogenesis ROS, PI3K-Akt signaling pathway, proteoglycans in cancer, and viral carcinogenesis (Figure 4). According to the proportions of each vertical setting as shown in Sankey diagram, luteolin mosloflavone I and mosloflavone II played a central role in the protection of MSTF on the IAV-induced lung inflammation in mice mostly through inhibiting pathways in cancer, MAPK signaling pathway, lipid and atherosclerosis, and chemical carcinogenesis ROS by targeting MAPK14, JAK2/3, HSP90, and HRAS, indicating the anti-inflammatory mechanism of MSTF.

### 3.5. Effects of MSTF on the Expressions of Key IAV-Mediated Proteins

**Figure 5:**
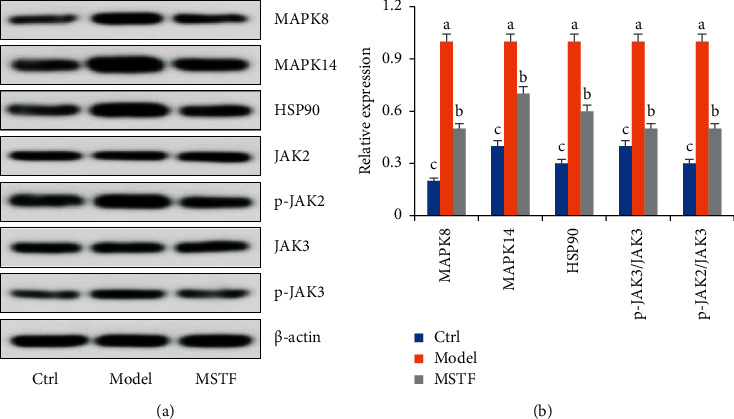
Effects of MSTF on the expressions of core targets in lung tissues of IAV-infected mice. (a) Representative results of protein blots. (b) Semiquantitative analysis of those core protein expressions. All data were shown as mean ± SEM (*n* = 5). Different letters meant significant differences (*P* < 0.05) by Tukey's test.

To confirm the correctness of network pharmacological findings, the expression profiles of five influenza-mediated proteins were measured by western blot analysis. Compared with those in the control group, the expressions of MAPK8, MAPK14, EGFR, p-JAK2, and PIK3R1 had increased 2-3-fold (Figure 5). However, MSTF intervention significantly suppressed the expressions of the five previously mentioned proteins, which were the key mediators of MAPK signaling pathway, chemical carcinogenesis ROS, and PI3K-Akt signaling pathway. It indicated that MSTF could protect mice against IAV-induced lung inflammation via inactivation of MAPK, PI3K-Akt, and ROS-related signaling pathways.

## 4. Discussion

Viral pneumonia is a common and frequent clinical disease caused by a variety of respiratory viruses, of which influenza virus is the most common pathogen, leading to a high mortality rate. The pathogenesis of viral pneumonia is thought to be twofold: first, direct damage to the respiratory organs caused by the invasion of the virus into the respiratory tract; second, the interaction between the body and the virus to produce an inflammatory response and immune damage [28–31]. Thus, inhibiting the key proteins involved in the host antiviral response could be a promising strategy for preventing various pneumonia. In this study, six flavonoids were identified from the traditional Chinese herb *M. scabra*, which was widely used as an antiviral and anti-inflammatory agent for years, and then based on the network pharmacology analysis and experimental verification, these flavonoids were demonstrated to protect against IAV-induced lung inflammation in mice mostly through inhibiting pathways in cancer, MAPK signaling pathway, lipid and atherosclerosis, and chemical carcinogenesis ROS.

In-silico techniques including network pharmacology analysis had been recommended to be the effective strategies for drug discovery and development. Docking enables the identification of novel compounds of therapeutic interest, predicting ligand-target interactions at a molecular level, or clarifying the structure-activity relationships [32–37]. It was especially suited for the assessing the overall therapeutic effects of herbal medicine, which always contained various components [38]. Recently, network pharmacology has been increasingly applied to reveal the molecular mechanisms of herbal formula [39–42]. In this study, the public service tools were employed to find the potential protein targets of five main flavonoids in MSTF, which could be divided into three clusters, namely, inflammation, proliferation, and apoptosis. It indicated that anti-inflammatory activity was one of the main protective effects of MSTF against IAV-induced lung injury.

The intrinsic immune system recognizes and clears exogenous pathogenic microorganisms and endogenous molecules released from the host cells through pattern recognition receptors (PRR) that recognize pathogen-associated molecular patterns (PAMP) or damage-associated molecular patterns (DAMP) [43–45]. The pulmonary macrophages have been important immune effector cells that literally engulf and destroy deformed cells and attack invaders, like bacteria or viruses [46, 47]. During various viral infection, macrophages recognize the PAMP of the influenza virus through the PRR, such as TLR7, RIG-I, and NLR, which initiates intracellular signaling and ultimately secretion of large amounts of proinflammatory cytokines, triggering an irresistible inflammatory storm [48–51]. The inflammatory response is the main mechanism of the body's intrinsic immune defense, but an excessive inflammatory response can lead to tissue damage and even systemic inflammatory response syndrome [45, 52]. Previous study had proved that MSTF ameliorated acute lung damage and cytokine storm in mice via repressing activation of TLR7-and RIG-I-dependent NF-*κ*B pathways [14]. Our study also found that MSTF exerted remarkedly anti-inflammatory effects on IAV-induced pulmonary edema and inflammatory cell infiltration. Interestingly, unlike the results of previous reports [14, 20], our findings suggested that pathways in cancer, MAPK signaling pathway, lipid and atherosclerosis, and chemical carcinogenesis ROS played a major role in performing anti-inflammatory effects of MSTF, which counted for more than 70% of total actions as shown in Sankey diagram. It indicated that, besides PRR-mediated pathways, MSTF not only inhibited the inflammation via inactivation of MAPK and JAK2/3 pathways but also inhibited proliferation and activation of inflammatory cells via suppressing cell proliferation-related genes, such as PIK3R1, MET, HRAS, and ERK2. However, these hypothetical inhibitions of MSTF on inflammatory cells including macrophages, and neutrophils should be further investigated. Therefore, this result provided a new perspective to reflect the pharmacological profiles of antiviral natural flavonoids.

TNF-*α* is the earliest and most important inflammatory mediator in the response to viral and bacterial infection. Under normal conditions, TNF-*α* activity is very low in the body, but it is secreted by mononuclear macrophages after influenza virus infection and binds to lung TNF-*α* receptors, causing lung injury by disrupting lysosomes and causing outflow of contents [53]. It is a proinflammatory cytokine that induces a cascade amplification of inflammation and induces the synthesis and secretion of IL-6. IL-6 is produced by a variety of cells, including lymphocytes and monocytes, and is also an important cytokine in the acute response period, inducing the adhesion and aggregation of inflammatory cells and causing pulmonary edema by increasing pulmonary vascular permeability, which is also a sign of activation of the cytokine cascade response [54, 55]. Moreover, IL-18 is important intracellular proinflammatory cytokine, mainly produced by monocytes and macrophages. When IAV infection occurs, monocytes and macrophages in the body accumulate in lung tissue and are activated; a large amount of IL-18 is matured and released outside the cells [56, 57]. IL-18 can induce the production of IFN-*γ* by immune cells, activate NK cells, stimulate *T* cell proliferation, enhance the cytotoxic effect of lymphocytes, and induce the production of IFN-*γ*, IL-2, GM-CSF, and other cytokines by Th1 cells, leading to inflammation [58–62]. In our study, we also found that those three proinflammatory cytokines were dramatically elevated after IAV challenge. Oral treatment with MSTF (50–200 mg/kg) could dose-dependently decrease the levels of TNF-*α*, IL-6, and IL-18 in the serum of IAV-infected mice. It indicated that MSTF reduced excessive inflammatory reaction brought by activated inflammatory cells in IAV-infected mice, thereby lightening cytokine storm-induced lung immune damage.

In conclusion, MSTF effectively reduced the levels of inflammatory cytokines and improved lung inflammation in IAV-infected mice. Notably, luteolin, kaempferol, apigenin, mosloflavone I, and mosloflavone II were the main effectors in MSTF, which primarily downregulated the expressions of targets MAPK14, MET, HRAS, HSP90, PIK3R1, and JAK2/3, and subsequently inhibited the activation of MAPK, JAK2/3, and PI3K/Akt pathways, thereby alleviating IAV-induced lung inflammation.

## Figures and Tables

**Table 1:** The ADME parameters of main compounds in total flavonoids from *Mosla scabra*.

No.	Compound	Name	OB (%)	DL	Caco-2 absorption	TPSA
1	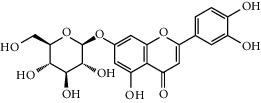	Luteoloside	7.29	−1.23	0.78	190.28 Å^2^

2	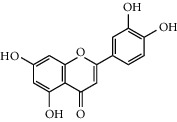	Luteolin	36.16	0.19	0.25	111.13 Å^2^

3	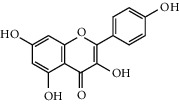	Kaempferol	41.88	0.26	0.24	111.13 Å^2^

4	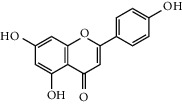	Apigenin	23.06	0.43	0.21	90.90 Å^2^

5	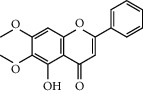	Mosloflavone I	34.04	0.86	0.26	68.90 Å^2^

6	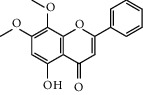	Mosloflavone II	44.09	1.01	0.25	68.90 Å^2^

ADME: absorption, distribution, metabolism, and excretion; OB: oral bioavailability; DL: drug-likeness; TPSA: topological polar surface area.

## Data Availability

The data used to support the findings of this study are included within the article.

## Abbreviations

DAMP: Damage-associated molecular patterns
DL: Drug-likeness
IAV: Influenza A virus
MSAE: Ethyl acetate extract of *M. scabra*
MSTF: Total flavonoids from *M. scabra*
OB: Oral bioavailability
PAMP: Pathogen-associated molecular patterns
PRR: Pattern recognition receptors
RF-PCR: Real time fluorescent quantitative PCR
ROS: Reactive oxygen species.
